# The impact of the Oakland sugar-sweetened beverage tax on price promotions of sugar-sweetened and alternative beverages

**DOI:** 10.1371/journal.pone.0285956

**Published:** 2023-06-09

**Authors:** Osama M. El-Sayed, Lisa M. Powell

**Affiliations:** Division of Health Policy and Administration, School of Public Health, University of Illinois Chicago, Chicago, Illinois, United States of America; Medical Research Council / Uganda Virus Research Institute & London School of Hygiene and Tropical Medicine Uganda Research Unit, UGANDA

## Abstract

The goal of sugar-sweetened beverage (SSB) taxes is to raise the prices of SSBs to decrease consumption. Price promotions play an important role in the sales of SSBs and could potentially be used by manufacturers to weaken the impact of such taxes. The purpose of this study is to determine how price promotions changed after the introduction of the 2017 Oakland SSB tax. A difference-in-differences study design was used to compare changes in prices and the prevalence and amount of price promotions for beverages in Oakland, California, relative to Sacramento, California, using two different datasets. Nielsen Retail Scanner data included price promotions for beverages sold and store audit data included price promotions offered by retailers. Changes were analyzed for SSBs, noncalorically sweetened beverages, and unsweetened beverages. After the implementation of the tax, the prevalence of price promotions for SSBs did not change significantly in Oakland relative to the comparison site of Sacramento. However, the depth of price promotions increased by an estimated 0.35 cents per ounce (P<0.001) based on the Nielsen retail scanner data and by 0.39 cents per ounce (P<0.001) based on the store audit data. This increase in the amount by which SSBs were price promoted following the introduction of the Oakland SSB tax may reflect a strategy by manufacturers to weaken the tax and/or retailers to bolster demand.

## Introduction

Diabetes is a major health problem in America and across the world. According to the 2022 National Diabetes Statistics Report, an estimated 14.7 percent of US adults have diabetes with another 38 percent being prediabetic [1]. This represents a major source of morbidity and mortality in America, with long-term outcomes of diabetes including chronic kidney disease, limb amputation, and heart disease. Dietary intervention remains the first-line treatment for patients with diabetes. Sugar-sweetened beverage (SSB) consumption is a risk factor for diabetes and obesity; and, in turn, obesity is a major risk factor for diabetes and cardiovascular and other noncommunicable diseases [2–4]. Thus, SSB dietary policy interventions are a key tool for obesity and diabetes prevention with SSBs representing the largest source of added sugars in the American diet [5,6].

Price promotions play an important role in the sales of SSBs. A price promotion is when a product is offered at a discounted price, often with an advertisement that indicates the reduced price. Manufacturers are motivated to offer price promotions in order to attract customers from competing brands and to accelerate purchases by customers [7]. One study from a large sample of products in the US showed that a disproportionate percentage of volume sold was sold during promotions [8]. It found that price promotions had a median lift factor—the ratio of volume sold on promotion to the volume sold off promotion—of 3.04. Recent evidence has shown that more frequent and deeper price promotions were associated with higher annual per capita SSB purchases [9]. One study found that an estimated 18.2 percent of SSBs were price-promoted in supermarkets and were among the most heavily price-promoted products food and beverage products [10]. Thus, price promotions play a role in encouraging obesogenic behavior through increasing consumption of SSBs.

It is for that reason that policymakers have become interested in targeting price promotions to address the obesity and diabetes epidemics, with some even considering overall bans on price promotions [11]. Opponents of restrictions on price promotions may argue that policies that involve restricting price promotions are an overreach of the role of government [12]. However, because of the negative externalities of SSB consumption and diabetes, this is an instance where the government’s role in promoting general welfare may take precedence. In other policy and product areas, Berkeley, California (CA), has already implemented a ban on unhealthy foods in checkout aisles and New York has implemented restrictions on the price promotions of tobacco products [13,14]. The potential impact of restricting price promotions of SSBs has been previously analyzed for Scotland and Australia. A study of Australia found that restricting SSB price promotions would be cost-effective, as the intervention would cost an estimated 17.0 million AUD but save 376.0 million AUD in healthcare-related expenses [15]. A study of Scotland found that restricting SSB price promotions could reduce annual sugar purchases by 9 percent [16]. To evaluate whether these restrictions on price promotions are warranted, it is valuable to first understand if and by how much companies may increase price promotions as a response to SSB taxes.

In 2017, Oakland, CA, implemented a one-cent-per-ounce SSB tax with a health goal of reducing sugar consumption [17]. It was found to be effective with an estimated increase in price of 0.49 cents per ounce and an estimated 14 percent decrease in volume sold as compared to the comparison of Sacramento in the first-year post-tax, although 46 percent of the decrease in volume sold was offset by cross-border shopping [18]. This effect increased in the second-year post-tax with an estimated increase in price of 0.67 cents per ounce and an estimated 18 percent decrease in volume sold as compared to Sacramento, with a net 6 percent decrease after accounting for cross-border shopping [19]. Both estimates for changes in price are for final price after any price promotions have been applied. Given the role that price-promotions play in SSB sales, it is possible that manufacturers may attempt to soften the impact of the tax by increasing price promotions post-tax implementation. This could help explain the less than full tax pass-through and would ultimately mean that the tax would be less effective at its intended effect of reducing demand for SSBs and their related adverse health consequences.

Keller et al previously looked at how SSB taxes impact marketing practices using Nielsen scanner data for five jurisdictions: Oakland, San Francisco, Philadelphia, Boulder, and Seattle [20]. Using a difference-in-differences (DID) model with distance-based matched control markets, in a working paper, they found a decrease in promotion frequency and a decrease in the relative promotion depth at 5-weeks post-tax. Two previous studies by Zenk et al examined how advertisements and in-store price promotions changed in the short-term and long-term after the Oakland SSB tax using store audit data. They found that the odds of price promotions for SSBs fell at 6-months post-tax, but there was no statistically significant change at 12- or 24-months post-tax [21,22]. In addition, change in the depth of sales for SSBs was not evaluated at 6- or 12-months post-tax and was not statistically significant at 24-months post-tax.

The purpose of this study is to investigate how price promotions changed after the introduction of the Oakland SSB tax over a two-year post-tax time period. In particular, this study intends to add to the literature by using a combination of retail scanner data and store audit data. The Nielsen data allow for analysis of the discount amount that consumers experienced on the purchases of the full spectrum of products sold at the sample of stores. Because the Nielsen data is on purchases, however, there is the potential that a larger discount may be explained by consumers purchasing products from different stores after the tax. For that reason, the store audit data allow for analysis of the discount amount offered by stores. This study examines changes in beverage prices and the prevalence and amount of price promotions in the full two-year post-tax period with a particular focus on changes in price promotion depth and emphasis on assessing whether changes may vary by neighborhood income of the store locations.

## Methods

### Data

#### Nielsen retail scanner data

This study uses Nielsen retail scanner data for Oakland and Sacramento, CA, from July 5, 2015, through June 29, 2019. As previously described in Leider et al [19], Sacramento was selected as a comparison site based on Mahalanobis matching performed on population size, the percent of the population that was non-Hispanic Black, the percent of the population that was Hispanic, median household income, the percent of the population below 125 percent of the poverty line, and the percent voting Democratic in the 2016 presidential election. In addition to the total dollar sales and total units sold for each universal product code (UPC), the Nielsen data also have information on the total dollar sales on price-promoted beverages and total units sold on price promotion for each UPC. These data are available for each week. Using the number of ounces per unit for each UPC, it was then possible to calculate the average final price, nonpromotional price, and promotional price by dividing the dollar sales by the ounces sold for all products, for nonpromoted products, and for promoted products. The discount amount was then calculated for each UPC by subtracting the promotional price from the nonpromotional price. This calculation represents the amount that purchased beverages were discounted. Because of the nature of the aggregated data, a change in average discount amount for consumers due to deeper discounts across all stores cannot be differentiated from consumers changing their distribution of purchases across stores in the sample. It is for that reason that this study also includes analyses on store audit data.

For analysis of the final price, nonpromotional price, promotional price, and discount amount, analysis was conducted on data from one-year pre- and two-years post-tax implementation with the year prior to the analytic period being used for weighting. The data from the year before the tax include dates from July 3, 2016 through July 1, 2017. The data from after the tax implementation date are from July 2, 2017 through June 29, 2019. Time pre- and post-tax implementation was aggregated into four-week time periods. These analyses were weighted using total volume sold from the year before the analysis (July 5, 2015 through July 2, 2016) as analytic weights.

The Nielsen data include a total of 11,638 UPCs. The UPCs include a combination of SSBs, noncalorically sweetened beverages (NSBs), and unsweetened beverages. Noncalorically sweetened beverages include beverages that are not sweetened with sugar but contain noncaloric sweetener. Unsweetened beverages included bottled water, 100% juice, and energy drinks, bottled coffee, and unsweetened sports drinks. Coding of the characteristics for UPCs were previously described [23]. The data were balanced on products that were available in each time-period. This removes any product that came into or fell out of the market over the study period. Balancing left a remaining 1,605 UPCs (887 SSBs, 228 NSBs, and 490 unsweetened beverages) which represent 83.5 percent of the total volume sold.

Table 1 contains summary statistics for the Nielsen Scanner data comparing prices and discount amount between Oakland and Sacramento. Beverages were categorized by size with individual-sized beverages being those that contain one liter or less per unit and family-sized beverages being those that are greater than one liter. Overall, nonpromotional prices and promotional prices tended to increase for all beverage types in both Oakland and Sacramento between the pre- and post-tax periods, but to a greater extent in Oakland for SSBs. The discount amount decreased between the pre- and post-tax period for each beverage type in Sacramento. The discount amount increased for SSBs in Oakland and stayed relatively the same for NSBs and unsweetened beverages.

**Table 1:** Nielsen scanner data summary statistics pre- and post-tax implementation.

	Sacramento, CA				Oakland, CA		
	Pre-tax Mean (95% CI)	Post-tax Mean (95% CI)	Difference		Pre-tax Mean (95% CI)	Post-tax Mean (95% CI)	Difference
**Nonpromotional Price (cents per ounce)**							
Sugar-sweetened Beverages (*n* = 887)	4.47 (4.4, 4.54)	4.52 (4.47, 4.57)	0.05		4.73 (4.66, 4.81)	5.53 (5.47, 5.58)	0.80
Individual sized (n = 466)	7.89 (7.75, 8.03)	7.96 (7.86, 8.06)	0.07		8.26 (8.11, 8.4)	9.18 (9.08, 9.29)	0.92
Family sized (n = 421)	3.09 (3.05, 3.13)	3.13 (3.1, 3.16)	0.04		3.31 (3.27, 3.35)	4.05 (4.02, 4.08)	0.74
Noncalorically Sweetened Beverages (n = 228)	5.06 (4.92, 5.2)	5.12 (5.02, 5.22)	0.06		5.54 (5.39, 5.68)	5.8 (5.69, 5.91)	0.26
Individual sized (n = 99)	9.3 (9.03, 9.57)	9.36 (9.17, 9.55)	0.06		9.89 (9.61, 10.16)	10.63 (10.42, 10.84)	0.74
Family sized (n = 129)	3.49 (3.41, 3.57)	3.55 (3.5, 3.61)	0.06		3.93 (3.85, 4.01)	4.01 (3.95, 4.07)	0.08
Unsweetened Beverages (n = 490)	3.08 (2.98, 3.17)	3.11 (3.04, 3.17)	0.03		3.22 (3.12, 3.32)	3.31 (3.24, 3.38)	0.09
Individual sized (n = 228)	8.29 (8.08, 8.49)	8.43 (8.29, 8.58)	0.14		8.59 (8.37, 8.81)	8.94 (8.79, 9.1)	0.35
Family sized (n = 262)	2.12 (2.04, 2.2)	2.13 (2.07, 2.18)	0.01		2.23 (2.15, 2.31)	2.27 (2.21, 2.33)	0.04
**Price Promotion Discount (cents per ounce)**							
Sugar-sweetened Beverages (n = 887)	0.78 (0.76, 0.8)	0.73 (0.71, 0.74)	-0.05		0.92 (0.9, 0.94)	1.22 (1.21, 1.24)	0.3
Individual sized (n = 466)	1.18 (1.15, 1.21)	1.11 (1.09, 1.14)	-0.07		1.25 (1.21, 1.28)	1.56 (1.54, 1.59)	0.31
Family sized (n = 421)	0.62 (0.6, 0.63)	0.57 (0.56, 0.58)	-0.05		0.78 (0.77, 0.8)	1.08 (1.07, 1.1)	0.3
Noncalorically Sweetened Beverages (n = 228)	0.82 (0.8, 0.84)	0.77 (0.75, 0.78)	-0.05		1.12 (1.09, 1.14)	1.13 (1.11, 1.16)	0.01
Individual sized (n = 99)	0.94 (0.9, 0.99)	0.83 (0.79, 0.86)	-0.11		1.23 (1.18, 1.28)	1.54 (1.49, 1.6)	0.31
Family sized (n = 129)	0.77 (0.75, 0.8)	0.74 (0.73, 0.76)	-0.03		1.08 (1.05, 1.11)	0.98 (0.96, 1)	-0.1
Unsweetened Beverages (n = 490)	0.52 (0.49, 0.54)	0.5 (0.48, 0.51)	-0.02		0.64 (0.61, 0.67)	0.63 (0.61, 0.65)	-0.01
Individual sized (n = 228)	1.31 (1.25, 1.38)	1.33 (1.29, 1.37)	0.02		1.55 (1.48, 1.63)	1.58 (1.53, 1.63)	0.03
Family sized (n = 262)	0.36 (0.34, 0.38)	0.33 (0.32, 0.34)	-0.03		0.47 (0.44, 0.49)	0.44 (0.43, 0.46)	-0.03
**Discount Proportion**							
Sugar-sweetened Beverages (n = 887)	0.19 (0.19, 0.19)	0.17 (0.17, 0.17)	-0.02		0.21 (0.21, 0.21)	0.24 (0.24, 0.24)	0.03
Individual sized (n = 466)	0.17 (0.17, 0.18)	0.17 (0.17, 0.17)	0		0.17 (0.16, 0.17)	0.19 (0.19, 0.19)	0.02
Family sized (n = 421)	0.19 (0.19, 0.2)	0.17 (0.17, 0.18)	-0.02		0.23 (0.22, 0.23)	0.26 (0.26, 0.26)	0.03
Noncalorically Sweetened Beverages (n = 228)	0.19 (0.19, 0.19)	0.18 (0.18, 0.18)	-0.01		0.24 (0.23, 0.24)	0.22 (0.22, 0.22)	-0.02
Individual sized (n = 99)	0.11 (0.11, 0.12)	0.1 (0.1, 0.11)	-0.01		0.14 (0.13, 0.14)	0.15 (0.14, 0.16)	0.01
Family sized (n = 129)	0.22 (0.21, 0.22)	0.21 (0.21, 0.21)	-0.01		0.27 (0.27, 0.28)	0.25 (0.24, 0.25)	-0.02
Unsweetened Beverages (n = 490)	0.15 (0.15, 0.16)	0.15 (0.14, 0.15)	0		0.19 (0.19, 0.2)	0.19 (0.18, 0.19)	0
Individual sized (n = 228)	0.16 (0.15, 0.16)	0.15 (0.15, 0.16)	-0.01		0.18 (0.18, 0.19)	0.17 (0.17, 0.17)	-0.01
Family sized (n = 262)	0.15 (0.15, 0.16)	0.14 (0.14, 0.15)	-0.01		0.19 (0.19, 0.2)	0.19 (0.19, 0.19)	0
**Promotional Price (cents per ounce)**							
Sugar-sweetened Beverages (n = 887)	3.7 (3.64, 3.77)	3.81 (3.76, 3.86)	0.11		3.82 (3.75, 3.89)	4.35 (4.3, 4.41)	0.53
Individual sized (n = 466)	6.7 (6.56, 6.84)	6.84 (6.74, 6.94)	0.14		7 (6.86, 7.15)	7.63 (7.52, 7.74)	0.63
Family sized (n = 421)	2.48 (2.45, 2.52)	2.57 (2.55, 2.6)	0.09		2.52 (2.49, 2.56)	2.99 (2.96, 3.02)	0.47
Noncalorically Sweetened Beverages (n = 228)	4.24 (4.1, 4.38)	4.37 (4.27, 4.47)	0.13		4.44 (4.3, 4.58)	4.66 (4.56, 4.77)	0.22
Individual sized (n = 99)	8.38 (8.11, 8.65)	8.56 (8.36, 8.75)	0.18		8.77 (8.49, 9.05)	9.21 (9.01, 9.42)	0.44
Family sized (n = 129)	2.71 (2.64, 2.78)	2.82 (2.77, 2.87)	0.11		2.84 (2.77, 2.91)	7.25 (7.11, 7.39)	4.41
Unsweetened Beverages (n = 490)	2.6 (2.51, 2.68)	2.72 (2.66, 2.78)	0.12		2.56 (2.47, 2.65)	2.74 (2.67, 2.81)	0.18
Individual sized (n = 228)	6.92 (6.73, 7.1)	7.09 (6.96, 7.21)	0.17		6.95 (6.75, 7.15)	3 (2.95, 3.05)	-3.95
Family sized (n = 262)	1.78 (1.71, 1.85)	1.85 (1.8, 1.91)	0.07		1.74 (1.67, 1.82)	1.84 (1.79, 1.9)	0.10

Values were computed for each UPC within each city pre- and post- tax implementation. The “discount proportion” refers to how much the price was discounted from the non-promoted price. The sample sizes include total number of UPCs. CI = confidence interval.

#### Store audit data

To test for heterogeneous effects by store type on the changes in nonpromotional price, promotional price, and discount amount, store audit data that were developed as part of a larger SSB tax evaluation project at the University of Illinois Chicago were used. Store audits were conducted with the Beverage Tax Food Store Observation Form, which included information on nonpromotional price and price promotion discounts and were previously described [22]. Reliability testing of the audit tool generally showed strong inter-rater reliability [24]. For the variables used in this study the kappa statistics were 0.80 or greater on average and the intraclass correlation coefficients were greater than 0.90. Seven store types were audited including general merchandise stores, supermarket, grocery stores, chain and non-chain convenience stores, drug stores, and small discount stores. Audits were conducted by trained auditors using the Beverage Tax Food Store Observation Form to log the prices and discounts at each visit. Stores were chosen for audit in each site by first randomizing 16 spatially balanced seed points on the maps of each site. The closest store to the seed points from each of the store types were then chosen for auditing. Stores were audited at baseline, 6 months post-tax, 12 months post-tax, and 24 months post-tax. The baseline audits were performed one month prior to tax implementation.

Altogether, 126 stores in Oakland and 124 stores in Sacramento were audited with 955 total audits conducted. The price promotion analytic sample was balanced for each store on products that were available at baseline and at least one other time period. The audit form included 59 taxed SSBs, 37 NSBs and 32 unsweetened beverages. After balancing, there were 33,214 available observations with products being price promoted in 8,539 observations. This balancing strategy removed observations for stores that did not offer the product both pre- and post-tax implementation. This balancing strategy differed from the Nielsen data because the audit tool contained more popular products. The Nielsen data, by contrast, contained all beverages sold and required balancing on products that were available at every time point in order to remove products that came into or fell out of the market.

Table 2 contains summary statistics for the Store Audit Data overall and by store type for final price, nonpromotional price, conditional discount, unconditional discount, and prevalence of promotion. The conditional discount amount is the amount of discount when there is a discount present while the unconditional discount amount is how much discounting decreased the price overall whether or not a sale was present. Analyses and summary statistics were weighted to reflect the distribution of volume sold using calculations based on the Nielsen data as previously described [22].

**Table 2:** Store audit data summary statistics pre- and post-tax implementation.

		Sacramento, CA				Oakland, CA		
		Pre-tax Mean (95% CI)	Post-tax Mean (95% CI)	Difference		Pre-tax Mean (95% CI)	Post-tax Mean (95% CI)	Difference
**Final Price (cents per ounce)**								
Sugar-sweetened Beverages		5.44 (5.27, 5.62)	5.53 (5.42, 5.65)	0.09		6.16 (5.91, 6.42)	6.83 (6.67, 6.99)	0.67
(n = 3,037; 7,792; 2,118; 4,663)								
Noncalorically Sweetened Beverages		5.38 (5.12, 5.65)	5.64 (5.46, 5.82)	0.26		5.78 (5.4, 6.16)	6.44 (6.18, 6.71)	0.66
(n = 1,577; 3,996; 915; 1,990)								
Unsweetened Beverages		4.88 (4.7, 5.06)	4.74 (4.63, 4.85)	-0.14		5.08 (4.82, 5.34)	5.01 (4.85, 5.17)	-0.07
(n = 1,265; 3,125; 873; 1,863)								
**Nonpromotional Price (cents per ounce) **								
Sugar-sweetened Beverages		6.08 (5.89, 6.27)	6.11 (5.99, 6.23)	0.03		6.87 (6.59, 7.14)	7.44 (7.27, 7.61)	0.57
(n = 3,037; 7,792; 2,118; 4,663)								
Noncalorically Sweetened Beverages		6.03 (5.74, 6.31)	6.23 (6.04, 6.41)	0.2		6.46 (6.05, 6.88)	6.92 (6.64, 7.19)	0.46
(n = 1,577; 3,996; 915; 1,990)								
Unsweetened Beverages		5.05 (4.86, 5.24)	4.9 (4.78, 5.02)	-0.15		5.29 (5.02, 5.56)	5.12 (4.96, 5.28)	-0.17
(n = 1,265; 3,125; 873; 1,863)								
**Conditional Discount Amount (cents per ounce) **								
Sugar-sweetened Beverages		1.66 (1.57, 1.74)	1.63 (1.57, 1.69)	-0.03		1.71 (1.57, 1.84)	1.99 (1.89, 2.08)	0.28
(n = 1,073; 2,286; 535; 937)								
Noncalorically Sweetened Beverages		1.67 (1.56, 1.77)	1.56 (1.49, 1.64)	-0.11		1.64 (1.48, 1.8)	1.75 (1.61, 1.89)	0.11
(n = 660; 1,359; 331; 523)								
Unsweetened Beverages		1.11 (0.96, 1.27)	1.19 (1.07, 1.31)	0.08		1.29 (1.03, 1.55)	1.12 (0.93, 1.31)	-0.17
(n = 175; 375; 101; 184)								
**Unconditional Discount Amount (cents per ounce)**								
Sugar-sweetened Beverages		0.58 (0.54, 0.62)	0.5 (0.48, 0.53)	-0.08		0.45 (0.4, 0.49)	0.48 (0.45, 0.51)	0.03
(n = 3,037; 7,792; 2,118; 4,663)								
Noncalorically Sweetened Beverages		0.62 (0.56, 0.68)	0.55 (0.52, 0.59)	-0.07		0.51 (0.44, 0.58)	0.43 (0.38, 0.48)	-0.08
(n = 1,577; 3,996; 915; 1,990)								
Unsweetened Beverages		0.14 (0.11, 0.17)	0.13 (0.11, 0.15)	-0.01		0.14 (0.1, 0.17)	0.08 (0.06, 0.11)	-0.06
(n = 1,265; 3,125; 873; 1,863)								
**Prevalence of Price Promotion**								
Sugar-sweetened Beverages		0.35 (0.34, 0.37)	0.29 (0.28, 0.3)	-0.06		0.25 (0.23, 0.27)	0.2 (0.19, 0.21)	-0.05
(n = 3,037; 7,792; 2,118; 4,663)								
Noncalorically Sweetened Beverages		0.42 (0.39, 0.44)	0.34 (0.33, 0.35)	-0.08		0.36 (0.33, 0.39)	0.26 (0.24, 0.28)	-0.1
(n = 1,577; 3,996; 915; 1,990)								
Unsweetened Beverages		0.14 (0.12, 0.16)	0.12 (0.11, 0.13)	-0.02		0.12 (0.09, 0.14)	0.1 (0.09, 0.11)	-0.02
(n = 1,265; 3,125; 873; 1,863)								

The conditional discount amount is the amount of discount when there is a discount present while the unconditional discount amount is how much discounting decreased the price overall whether or not a sale was present. The prevalence of price promotion represents the proportion of observations that had a sale present for each product. The sample size includes total number of observations for Sacramento pre-tax, Sacramento post-tax, Oakland pre-tax, and Oakland post-tax respectively. CI = confidence interval.

To summarize, Table 3 lists the outcome variables that are evaluated in the retail scanner and store audit datasets with their descriptions.

**Table 3:** Outcome variables used in each dataset.

	Variable	Description
**Retail Scanner Data Variables**		
	Nonpromotional Price	The mean price for each UPC when a price promotion was not present
	Promotional Price	The mean price for each UPC when a price promotion was present
	Discount Amount	The difference between the mean nonpromotional price and the mean promotional price
	Final Price	The overall mean price—whether promotional or nonpromotional—for each UPC at each time period
**Store Audit Data Variables**		
	Nonpromotional Price	The price of each product when a price promotion was not present
	Promotional Price	The price of each product when a price promotion was present
	Discount Amount	The amount the price was reduced when it was price promoted. The change in discount amount was estimated conditional on a price promotion being offered and unconditionally.
	Final Price	The price—promotional or nonpromotional—of each product at each store
	Prevalence of Promotion	The proportion of stores that were offering a price promotion for each product at each time period

A summary of the outcome variables analyzed in the retail scanner and store audit datasets.

### Models

#### Nielsen retail scanner data

In order to estimate how nonpromotional price, promotional price, and amount of discount changed after tax implementation, DID models were used. The linear regression equation for the DID model is:

$$\begin{array}{c} Outcome_{ict}=\beta_{0}+\beta_{1}Oakland_{c}+\beta_{2}Post_{t}*Oakland_{c}+\lambda_{i}+\delta_{t}+\epsilon_{ict} \end{array}$$

where i represents the UPC, c represents the city, and t represents the time-period. Post_t_ is an indicator for whether the observation takes place after the implementation date, and Oakland_c_ is an indicator for whether the observation takes place in the city of Oakland. The parameter for the interaction between Post_t_ and Oakland_c_, β_2_, represents the DID estimate for the impact of the tax on the given outcome. This approach includes individual-UPC fixed effects, represented by λ_i_, and time-period fixed effects represented by δ_t_. Models were run with robust standard errors for each beverage type (SSB, NSB, and unsweetened beverages) and for individual-sized vs family-sized products within each beverage type. For each UPC, volume sold from the year prior to the analytic sample was used to weight the analysis. Due to multiple comparisons, a Holm-Bonferroni correction was performed for each outcome variable.

To evaluate the parallel trends assumption for DID analyses, S1–S6 Figs show the trends in nonpromotional price and depth of price promotion over time in Oakland and Sacramento for each beverage type. As can be seen visually, the nonpromotional price and depth of price promotion follow a similar trend for one year pre-tax implementation for each beverage type. In addition, an event study model (S1 File and S7 Fig) is included to evaluate how the discount amount changed over time and provides evidence supporting that the pre-tax parallel trends assumption holds.

#### Store audit data

A DID model was used to analyze the change in final price, nonpromotional price, discount amount conditional on a sale being present, unconditional discount amount, and prevalence of promotion. The linear regression equation for the DID model is:

$$\begin{array}{c} Outcome_{icts}=\beta_{0}+\beta_{1}Oakland_{c}+\beta_{2}Post_{t}*Oakland_{c}+\lambda_{i}+\delta_{t}+\gamma_{s}+\epsilon_{icts} \end{array}$$

where i represents the product, c represents the city, t represents the time-period, and s represents the store. Post_t_ is an indicator for whether the observation takes place after the implementation date, and Oakland_c_ is an indicator for whether the observation takes place in the city of Oakland. The parameter for the interaction between Post_t_ and Oakland_c_, β_2_, represents the DID estimate for the impact of the tax on the given outcome. This approach includes individual-product fixed effects represented by λ_i_, time-period fixed effects represented by δ_t_, store fixed effects represented by *γ_s_*, and robust standard errors. This model is similar to the DID models for the Nielsen Retail Scanner data but also includes store-level fixed effects. Models were run with robust standard errors and with weights calculated using volume sold from the Nielsen data.

For the store audit data, models were estimated for stores from low-income, medium-income, and high-income areas separately. Cut-offs were determined using percentiles of median household income by census tract as determined by the American Community Survey [25]. Low-income areas were determined to be those that had a median household income of 37,526 dollars and below, which corresponds to the 33^rd^ percentile within the data. High-income areas were determined to be those that had a median household income greater than 59,531 dollars which corresponds to the 67^th^ percentile within the data. Medium-income areas were determined as those between low- and high-income median household income cutoffs (37,526 dollars to 59,531 dollars). Due to multiple comparisons, a Holm-Bonferroni correction was performed for each outcome variable.

This study did not involve human or animal participants, nor did the study take place on any private or protected areas. The study was approved and deemed exempt by the University of Illinois Chicago Institutional Review Board.

## Results

### Nielsen retail scanner data

Table 4 provides the retail scanner data DID estimates for changes in nonpromotional price, promotional price, and discount amount. After correcting for multiple comparisons using the Holm-Bonferroni procedure, the estimates with a p-value less than 0.01 remained significant at an alpha level of 0.05. For SSBs overall, the nonpromotional price was estimated to have increased by 0.75 cents per ounce (p<0.001) post-tax and the discount amount was estimated to have increased by 0.35 cents per ounce (p<0.001). Given the average pre-tax nonpromotional price of SSBs in Oakland was 4.73 cents per ounce, the estimate for the nonpromotional price represents an estimated 15.9 percent increase. Given the average pre-tax price promotion discount was 0.92 cents per ounce, the estimate for the discount amount represents a deepening of 38.0 percent. The individual-sized beverages had a slightly greater change in nonpromotional price and discount amount at 0.87 cents per ounce (p<0.001) and 0.39 cents per ounce (p<0.001), respectively, compared to 0.70 cents per ounce (p<0.001) and 0.34 cents per ounce (p<0.001), respectively, for family-sized SSBs.

**Table 4:** Retail scanner data difference-in-differences estimates for nonpromotional price, discount amount, promotional price, and percent sold on price promotion.

	Nonpromotional Price (cents per ounce)	Discount (cents per ounce)	Promotional Price (cents per ounce)
Sugar-sweetened Beverages (n = 887)	0.75*** (0.72, 0.77)	0.35*** (0.32, 0.38)	0.41*** (0.38, 0.43)
Individual sized (n = 466)	0.87*** (0.82, 0.92)	0.39*** (0.33, 0.45)	0.5*** (0.44, 0.56)
Family sized (n = 421)	0.7*** (0.67, 0.72)	0.34*** (0.31, 0.37)	0.37*** (0.34, 0.39)
Noncalorically Sweetened Beverages (n = 228)	0.2*** (0.16, 0.24)	0.07*** (0.03, 0.11)	0.13*** (0.09, 0.16)
Individual sized (n = 99)	0.69*** (0.61, 0.77)	0.44*** (0.34, 0.53)	0.27*** (0.19, 0.35)
Family sized (n = 129)	0.02 (-0.02, 0.05)	-0.06** (-0.1, -0.02)	0.07*** (0.05, 0.1)
Unsweetened Beverages (n = 490)	0.06*** (0.04, 0.08)	0.01 (-0.02, 0.04)	0.05*** (0.02, 0.08)
Individual sized (n = 228)	0.21*** (0.14, 0.28)	0.03 (-0.08, 0.15)	0.18*** (0.08, 0.27)
Family sized (n = 262)	0.03** (0.01, 0.05)	0.01 (-0.02, 0.04)	0.02 (0, 0.05)

Linear regression difference-in-differences models compared changes in nonpromotional prices, discount amount, and promotional prices in Oakland compared to Sacramento. Sample sizes of UPCs for each beverage type are included in parentheses next to the category and 95% confidence intervals are included in parentheses next to the estimates. After correcting for multiple comparisons using the Holm-Bonferroni procedure, those estimates with a p-value less than 0.01 remained significant at an alpha level of 0.05. * p<0.05; ** p<0.01; *** p<0.001.

The nonpromotional price of NSBs overall was estimated to have increased by 0.20 cents per ounce (p<0.001) with the amount of discount increasing by 0.07 cents per ounce (p<0.001). This change was driven by a change in the nonpromotional price of individual-sized NSBs of 0.69 cents per ounce (p<0.001) and change in discount amount of 0.44 cents per ounce (p<0.001). The nonpromotional price of unsweetened beverages was estimated to have increased by only 0.06 cents per ounce (p<0.001) while the amount of discount did not change significantly. This increase was driven by an increase in the nonpromotional price of individual-sized unsweetened beverages of 0.21 cents per ounce (p<0.001).

### Store audit data

Table 5 includes the DID estimates for changes in final price, nonpromotional price, conditional and unconditional discount amount, and prevalence of promotion by store area income level. After correcting for multiple comparisons using the Holm-Bonferroni procedure, the estimates with a p-value less than 0.01 remained significant at an alpha level of 0.05. Overall, nonpromotional price increased for SSBs by an estimated 0.76 cents per ounce (p<0.001). The increase in the discount amount conditional on sale for SSBs was 0.39 cents per ounce (p<0.001).

**Table 5:** Store audit difference-in-differences estimates for changes in final price, nonpromotional price, discount amount, and prevalence of promotion by area income level.

	All	Low-Income	Medium-Income	High-Income
**Final Price**				
Sugar-sweetened Beverages (n = 17,610; 6,189; 5,977; 5,444)	0.68*** (0.55, 0.8)	0.76*** (0.5, 1.03)	0.49*** (0.3, 0.69)	0.82*** (0.6, 1.03)
Noncalorically Sweetened Beverages (n = 8,478; 2,593; 2,811; 3,074)	0.38*** (0.25, 0.5)	0.6*** (0.32, 0.88)	0.24* (0.05, 0.43)	0.45*** (0.24, 0.67)
Unsweetened Beverages (n = 7,126; 2,379; 2,313; 2,434)	0.08 (-0.03, 0.19)	0.05 (-0.16, 0.26)	0.14 (-0.05, 0.32)	0.08 (-0.11, 0.26)
**Nonpromotional Price**				
Sugar-sweetened Beverages (n = 17,610; 6,189; 5,977; 5,444)	0.76*** (0.65, 0.86)	0.76*** (0.55, 0.98)	0.69*** (0.52, 0.86)	0.83*** (0.69, 0.98)
Noncalorically Sweetened Beverages (n = 8,478; 2,593; 2,811; 3,074)	0.39*** (0.29, 0.48)	0.49*** (0.3, 0.68)	0.46*** (0.31, 0.61)	0.3*** (0.15, 0.45)
Unsweetened Beverages (n = 7,126; 2,379; 2,313; 2,434)	0.01 (-0.09, 0.11)	-0.08 (-0.26, 0.11)	0.2* (0.02, 0.38)	-0.07 (-0.23, 0.08)
**Conditional Discount Amount**				
Sugar-sweetened Beverages (n = 4,831; 1,335; 1,551; 1,945)	0.39*** (0.21, 0.58)	0.61* (0.05, 1.17)	0.53*** (0.23, 0.83)	0.19 (-0.05, 0.42)
Noncalorically Sweetened Beverages (n = 2,873; 787; 895; 1,191)	0.16 (-0.01, 0.33)	-0.09 (-0.44, 0.26)	0.22 (-0.06, 0.51)	0.13 (-0.11, 0.38)
Unsweetened Beverages (n = 835; 218; 273; 344)	-0.22 (-0.59, 0.15)	-0.64 (-1.34, 0.07)	0.56 (-0.79, 1.9)	-0.41 (-0.82, 0)
**Unconditional Discount Amount**				
Sugar-sweetened Beverages (n = 17,610; 6,189; 5,977; 5,444)	0.11* (0.01, 0.21)	0.1 (-0.15, 0.34)	0.23** (0.09, 0.37)	0.01 (-0.16, 0.19)
Noncalorically Sweetened Beverages (n = 8,478; 2,593; 2,811; 3,074)	0 (-0.1, 0.1)	-0.13 (-0.36, 0.1)	0.17* (0.01, 0.32)	-0.12 (-0.29, 0.04)
Unsweetened Beverages (n = 7,126; 2,379; 2,313; 2,434)	-0.07* (-0.14, 0)	-0.12 (-0.25, 0.01)	0.05 (-0.05, 0.15)	-0.14* (-0.26, -0.02)
**Prevalence of Promotion**				
Sugar-sweetened Beverages (n = 17,610; 6,189; 5,977; 5,444)	-0.03 (-0.07, 0.01)	-0.02 (-0.09, 0.05)	-0.01 (-0.08, 0.05)	-0.06 (-0.13, 0.01)
Noncalorically Sweetened Beverages (n = 8,478; 2,593; 2,811; 3,074)	-0.09*** (-0.14, -0.04)	-0.04 (-0.15, 0.06)	-0.07 (-0.16, 0.01)	-0.14*** (-0.22, -0.07)
Unsweetened Beverages (n = 7,126; 2,379; 2,313; 2,434)	-0.02 (-0.05, 0.02)	-0.03 (-0.08, 0.02)	0.05* (0, 0.11)	-0.07* (-0.14, 0)

Linear regression difference-in-differences models compared changes in final price, nonpromotional prices, conditional discount amount, unconditional discount amount, and prevalence of promotion in Oakland to Sacramento. The conditional discount amount is the amount of discount on products when there was a discount present while the unconditional discount amount is how much discounting decreased the price overall unconditional on the presence of a sale. The prevalence of promotion represents the proportion of observations that had a sale present for each product. Sample sizes of each beverage type are included in parentheses next to the category and 95% confidence intervals are included in parentheses next to the estimates. After correcting for multiple comparisons using the Holm-Bonferroni method, those estimates with a p-value less than 0.01 remained significant at an alpha level of 0.05. * p<0.05; ** p<0.01; *** p<0.001.

Analyses by area income of the store locations, showed that the estimate for the discount amount conditional on sale for SSBs was the greatest in the low-income areas followed by the medium-income areas with estimates of 0.61 (P<0.05) and 0.53 (P<0.001) respectively. The estimate for the discount amount conditional on sale was not significant in high-income areas. The estimate for the conditional discount amount in the low-income area was not significant after correcting for multiple comparisons. Wald tests comparing the low-income and medium-income area parameters to the high-income parameter were not significant.

Overall, the prevalence of promotion did not change significantly for SSBs at any store area income level. The prevalence of promotion decreased for NSBs in high-income areas by 14 percentage points (P<0.001) but did not change significantly at stores in low- or medium-income areas. The prevalence of promotion decreased for unsweetened beverages in high-income areas by 7 percentage points (P<0.05) and increased in medium-income areas by 5 percentage points (P<0.05); both were not significant after correcting for multiple comparisons. The prevalence of promotion did not change significantly for unsweetened beverages in low-income areas significantly.

## Discussion

Overall, this study found that price promotion discount amounts increased for SSBs in both the retail scanner and store audit data after the implementation of the Oakland SSB tax with the store audit data showing that the prevalence of price promotions did not change significantly for SSBs. The estimated increase in the discount amount was found to be similar across the two data sets: 0.35 cents per ounce (P<0.001) based on the Nielsen retail scanner data and 0.39 cents per ounce (P<0.001) based on the store audit data. Taken together, this estimated increase in discount amount points to a possible strategy by manufacturers to increase the depth of price promotions in order to counteract the impact of the tax.

The way that price promotions are generally determined in consumer packaged goods (CPG) is by manufacturers allocating a set amount of money to be used for price promotions. This amount is referred to as the “trade spend” and it represents the second largest category of CPG manufacturer expenditures after cost of goods, averaging 10 to 25 percent of manufacturer revenue [26]. After the trade spend is set, the manufacturers will negotiate with retailers to determine how best to use the trade spend towards price promotions. In this process, the manufacturers pay the retailers to offer the price promotions.

When considering the area-income level of the stores, the estimates for discounts conditional on a price promotion being present were 0.61 cents per ounce (P<0.05) and 0.53 cents per ounce (P<0.001) for low- and medium-income areas respectively and 0.19 cents per ounce (not significant) for high-income areas. However, the Wald tests between these parameters were not significant across all income group comparisons. While a statistically significant difference across income levels could not be confirmed, the results suggest that manufacturers may have targeted lower to middle-income areas with this strategy over higher-income areas. This would also mean that the tax’s impact towards improving rates of diabetes and obesity is blunted to a greater extent in these lower to middle-income areas. This has important implications for health equity. Given the higher prevalence of obesity and diabetes in low-income areas [27,28], it is important to consider the role of the social determinants of health when designing policies for obesity and diabetes. This is particularly important given the evidence of targeted marketing towards underserved populations [29,30]. If a targeted manufacturer price promotion strategy did exist, it would suggest that a ban on price promotions may provide progressive benefits for communities with lower to middle incomes. Future studies should consider the role that store area income level plays in setting price promotions after SSB tax implementation in order to better address these health equity concerns.

From a policy perspective, because the depth of price promotions was found to have increased after the introduction of the SSB tax, there is a potential synergy between SSB tax policy and policies that restrict the price promotions of SSBs. If manufacturers were unable to respond to the tax with increased price promotions as they may have, it is possible that there would be a greater net pass through of SSB taxes to final prices. For that reason, more comprehensive policy strategies may have a multiplicative impact on preventing diabetes and obesity.

For NSBs, the results generally showed limited changes overall in the discount amount: based on the Nielsen scanner data, the estimated change in discount amount was 0.07 cents per ounce (P<0.001), and while the estimated change in the discount amount based on the store audit data was 0.16 cents per ounce, that estimate was not statistically significant. The estimates between the two datasets were similar. The slight discrepancy may be due to the differences in sample of stores, the sample of products, or the fact that the Nielsen data represent purchases while the store audit data represents shelf prices.

Previous studies that have examined changes in price promotions following the implementation of SSB taxes drawing on store audit and scanner data with differing post-tax follow up periods have found mixed results. In terms of the frequency of price promotions, Keller et al found a significant decrease in promotions at 5-weeks post tax implementation across five taxing jurisdictions based on scanner data [20], whereas Zenk et al. found a decrease in prevalence at 6-months and no changes in at 12- or 24-months in Oakland based on store audit data [21,22]. This study similarly found no change in prevalence of price promotions, on average, in the 2-years post-tax period in Oakland. More limited work has been done to assess changes in the depth of price promotions, which is important to understand the extent to which manufacturers may try to offset the net pass-through amount of the tax to prices faced by consumers. At 5-weeks post-tax, Keller et al. found a decrease in the promotion depth relative to the regular price, whereas Zenk et al. found an increase in the price promotion amount at 2-years post tax but it was not statistically significant. This study has added to the literature by assessing the depth of the price promotions over the entire two-year post-tax period and found consistent evidence from both the scanner and store audit data that, on average, the discount amount increased by 0.35 to 0.39 cents per ounce. This study further added to the literature by examining differences in price promotion outcomes based on store area income levels with findings that suggest that there may be some targeting of lower- to middle-income areas which deserves further investigation in future work.

There are a few limitations to note for this study. The first is that only one comparison city was used to estimate the effect. More precision could be achieved by adding additional comparison cities. Additionally, each dataset used had its own limitations. It was not possible to analyze the effect of retailers in the retail scanner data and the store audit data contained a limited number of sampled beverage products. It was for that reason that both datasets were analyzed in this study to get a better picture of how price promotions changed after the tax implementation.

Overall, this study found that the depth of price promotions for SSBs increased in Oakland after the introduction of the SSB tax. This was larger in stores in low- and medium- income areas than high-income area, though the differences were not statistically significant. This increase in price promotions may reflect a strategy by manufacturers to counteract the impact of the tax. Future evaluations of SSB tax impacts should monitor changes is price promotions to help inform policymakers on the extent to which marketing behaviors may, in part, undermine tax impacts.

## Supporting information

S1 Fig Nielsen scanner data trend in nonpromotional price for sugar-sweetened beverages. Each point represents a 4-week time period. July 1, 2017, the date of tax implementation, is represented by the dashed line.
(TIF)

S2 Fig Nielsen scanner data trend in discount amount for sugar-sweetened beverages. Each point represents a 4-week time period. July 1, 2017, the date of tax implementation, is represented by the dashed line.
(TIF)

S3 Fig Nielsen scanner data trend in nonpromotional price for noncalorically sweetened beverages. Each point represents a 4-week time period. July 1, 2017, the date of tax implementation, is represented by the dashed line.
(TIF)

S4 Fig Nielsen scanner data trend in discount amount for noncalorically sweetened beverages. Each point represents a 4-week time period. July 1, 2017, the date of tax implementation, is represented by the dashed line.
(TIF)

S5 Fig Nielsen scanner data trend in nonpromotional price for unsweetened beverages. Each point represents a 4-week time period. July 1, 2017, the date of tax implementation, is represented by the dashed line.
(TIF)

S6 Fig Nielsen scanner data trend in discount amount for unsweetened beverages. Each point represents a 4-week time period. July 1, 2017, the date of tax implementation, is represented by the dashed line.
(TIF)

S7 Fig Event study for change in final price, nonpromotional price, and discount amount of sugar-sweetened beverages. Each point represents an estimate from one time period from the event study approach. Time period 13 (June 4, 2017 –July 1, 2017) was used as the reference month and is represented in green. Bars represent the 95% confidence intervals. The solid line represents the tax implementation date.
(TIF)

S1 Table Analytical dataset description. (DOCX)

S1 Dataset Analytical dataset. (CSV)

S1 File Event study model description for change in final price, nonpromotional price, and discount amount of sugar-sweetened beverages. (DOCX)

## References

[1] Centers for Disease Control and Prevention. National Diabetes Statistics Report. 2022 June 29 [cited 2022 March 6]. Available from: https://www.cdc.gov/diabetes/data/statistics-report/index.html.

[2] Barnes AS. The epidemic of obesity and diabetes: trends and treatments. Tex Heart Inst J. 2011;38(2):142–4. ; PubMed Central PMCID: PMC3066828. 21494521 PMC3066828

[3] Faruque S, Tong J, Lacmanovic V, Agbonghae C, Minaya DM, Czaja K. The Dose Makes the Poison: Sugar and Obesity in the United States—a Review. Polish journal of food and nutrition sciences. 2019;69(3):219–33. doi: 10.31883/pjfns/110735 . 31938015 PMC6959843

[4] Meng Y, Li S, Khan J, Dai Z, Li C, Hu X, et al. Sugar- and Artificially Sweetened Beverages Consumption Linked to Type 2 Diabetes, Cardiovascular Diseases, and All-Cause Mortality: A Systematic Review and Dose-Response Meta-Analysis of Prospective Cohort Studies. Nutrients. 2021;13(8). Epub 2021/07/30. doi: 10.3390/nu13082636 ; PubMed Central PMCID: PMC8402166. 34444794 PMC8402166

[5] Hu FB, Malik VS. Sugar-sweetened beverages and risk of obesity and type 2 diabetes: epidemiologic evidence. Physiol Behav. 2010;100(1):47–54. Epub 2010/02/09. doi: 10.1016/j.physbeh.2010.01.036 ; PubMed Central PMCID: PMC2862460. 20138901 PMC2862460

[6] Bowman JC S., Friday J., LaComb R., Paudel D., Shimizu M. Added sugars in adults’ diet: what we eat in America, NHANES 2015–2016. Food Surveys Research Group. USDA-ARS. 2019 [Cited 2023 April 7]. Available from: https://www.ars.usda.gov/ARSUserFiles/80400530/pdf/DBrief/24_Sources_of_Added_Sugars_in_Adults%27_Diet_2015-2016.pdf.

[7] Anderson ET, Fox EJ. Chapter 9—How price promotions work: A review of practice and theory. In: Dubé J-P, Rossi PE, editors. Handbook of the Economics of Marketing. 1: North-Holland; 2019. p. 497–552.

[8] Hitsch GJ, Hortaçsu A, Lin X. Prices and promotions in U.S. retail markets. Quantitative Marketing and Economics. 2021;19(3):289–368. doi: 10.1007/s11129-021-09238-x

[9] Zhong Y, Auchincloss AH, Stehr MF, Langellier BA. Are price discounts on sugar-sweetened beverages (SSB) linked to household SSB purchases?—a cross-sectional study in a large US household and retail scanner database. Nutr J. 2021;20(1):29. Epub 2021/03/21. doi: 10.1186/s12937-021-00673-w PubMed Central PMCID: PMC7980678. 33740986 PMC7980678

[10] Powell LM, Kumanyika SK, Isgor Z, Rimkus L, Zenk SN, Chaloupka FJ. Price promotions for food and beverage products in a nationwide sample of food stores. Prev Med. 2016;86:106–13. Epub 2016/02/02. doi: 10.1016/j.ypmed.2016.01.011 . 26827618

[11] Krieger J, Bleich SN, Scarmo S, Ng SW. Sugar-Sweetened Beverage Reduction Policies: Progress and Promise. Annual Review of Public Health. 2021;42(1):439–61. doi: 10.1146/annurev-publhealth-090419-103005 33256536

[12] Herrin C. Berkeley Bans Unhealthy Foods from Grocery Store Checkout Lines. 2020 May 18, 2022. The Heartland Institute. 2020 October 2 [Cited 2022 December 17]. Available from: https://www.heartland.org/news-opinion/news/berkeley-bans-unhealthy-foods-from-grocery-store-checkout-lines.

[13] NYS Tobacco Control Policies. Department of Health. 2022 November [Cited 2022 December 17]. Available from: https://www.health.ny.gov/prevention/tobacco_control/current_policies.htm.

[14] Berekeley CA. Healthy Checkout, Stat. 9.82. 2020. Available from: https://berkeley.municipal.codes/BMC/9.82.

[15] Huse O, Ananthapavan J, Sacks G, Cameron A, Zorbas C, Peeters A, et al. The potential cost-effectiveness of mandatory restrictions on price promotions for sugar-sweetened beverages in Australia. International Journal of Obesity. 2020;44. doi: 10.1038/s41366-019-0495-9 31792336

[16] Dogbe W, Revoredo-Giha C. Industry levy versus banning promotion on soft drinks in Scotland: A distributional analysis. Food Policy. 2022;106:102191. doi: doi.org/10.1016/j.foodpol.2021.102191

[17] Sugar Sweetened Beverage Tax Frequently-Asked-Questions (FAQs). City of Oakland. 2017 December 30 [Cited 2022 December 17]. Available from: https://cao-94612.s3.amazonaws.com/documents/SSBT-Frequently-Asked-Questions-v2a.pdf.

[18] Léger PT, Powell LM. The impact of the Oakland SSB tax on prices and volume sold: A study of intended and unintended consequences. Health Econ. 2021;30(8):1745–71. Epub 2021/05/02. doi: 10.1002/hec.4267 . 33931915

[19] Leider J, Powell LM. Longer-term impacts of the Oakland, California, sugar-sweetened beverage tax on prices and volume sold at two-years post-tax. Soc Sci Med. 2021:114537. Epub 2021/11/29. doi: 10.1016/j.socscimed.2021.114537 . 34838326

[20] Keller KO, Guyt JY, Grewal R. Soda Taxes and Marketing Conduct. Kenan Institute of Private Enterprise. 2022. doi: 10.2139/ssrn.4127264

[21] Zenk SN, Li Y, Leider J, Pipito AA, Powell LM. No long-term store marketing changes following sugar-sweetened beverage tax implementation: Oakland, California. Health Place. 2021;68:102512. Epub 2021/02/01. doi: 10.1016/j.healthplace.2021.102512 . 33517072

[22] Zenk SN, Leider J, Pugach O, Pipito AA, Powell LM. Changes in Beverage Marketing at Stores Following the Oakland Sugar-Sweetened Beverage Tax. American Journal of Preventive Medicine. 2020;58(5):648–56. doi: 10.1016/j.amepre.2019.12.014 32192801

[23] Powell LM, Leider J. The impact of Seattle’s Sweetened Beverage Tax on beverage prices and volume sold. Econ Hum Biol. 2020;37:100856. Epub 2020/02/20. doi: 10.1016/j.ehb.2020.100856 . 32070906

[24] Li Y, Leider J, Pipito AA, Pugach O, Zenk SN, Powell LM. Development and Reliability Testing of a Food Store Observation Form for Use in Beverage Tax Evaluations. Illinois Prevention Research Center. 2018 December [Cited 2022 December 17]. Available from: https://p3rc.uic.edu/wp-content/uploads/sites/561/2019/11/Development-Reliability-Testing-of-Food-Store-Observation-Form_Illinois-PRC_Brief-No-108.pdf.

[25] U.S. Census Bureau 2011–2015 American Community Survey 5-year estimates. 2016 December 2 [Cited 2022 December 17]. Available from: https://www2.census.gov/programs-surveys/acs/summary_file/2015/data/5_year_by_state/.

[26] Gómez MI, Rao VR, McLaughlin EW. Empirical Analysis of Budget and Allocation of Trade Promotions in the U.S. Supermarket Industry. Journal of Marketing Research. 2007;44(3):410–24. doi: 10.1509/jmkr.44.3.410

[27] Gaskin DJ, Thorpe RJ Jr., McGinty EE, Bower K, Rohde C, Young JH, et al. Disparities in diabetes: the nexus of race, poverty, and place. American journal of public health. 2014;104(11):2147–55. Epub 2013/11/14. doi: 10.2105/AJPH.2013.301420 . 24228660 PMC4021012

[28] Ludwig J, Sanbonmatsu L, Gennetian L, Adam E, Duncan GJ, Katz LF, et al. Neighborhoods, obesity, and diabetes—a randomized social experiment. The New England journal of medicine. 2011;365(16):1509–19. doi: 10.1056/NEJMsa1103216 . 22010917 PMC3410541

[29] Powell LM, Wada R, Kumanyika SK. Racial/ethnic and income disparities in child and adolescent exposure to food and beverage television ads across the US media markets. Health & place. 2014;29:124–31. doi: 10.1016/j.healthplace.2014.06.006 25086271 PMC4501500

[30] Herrera AL, Pasch KE. Targeting Hispanic adolescents with outdoor food & beverage advertising around schools. Ethnicity & Health. 2018;23(6):691–702. doi: 10.1080/13557858.2017.1290217 28277028 PMC6091513

