# Supplementary figures and images for: The impact of the Oakland sugar-sweetened beverage tax on price promotions of sugar-sweetened and alternative beverages
Source: PLoS One. 2023 Jun 9;18(6):e0285956. doi: 10.1371/journal.pone.0285956 (PMC10256178; doi:10.1371/journal.pone.0285956)

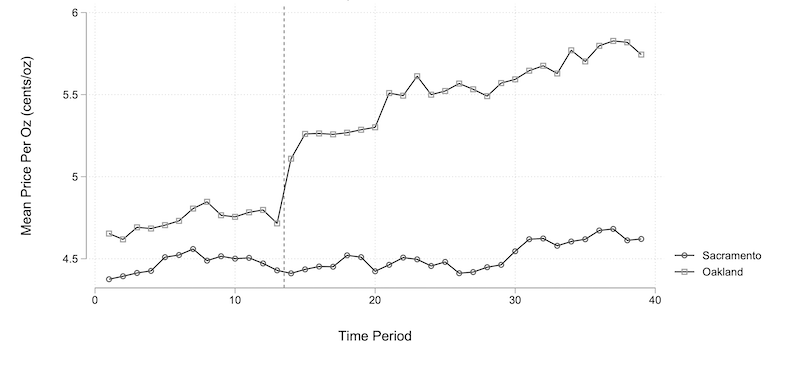

Supplement: S1 Fig — Each point represents a 4-week time period. July 1, 2017, the date of tax implementation, is represented by the dashed line. (TIF) [file pone.0285956.s001.tif]

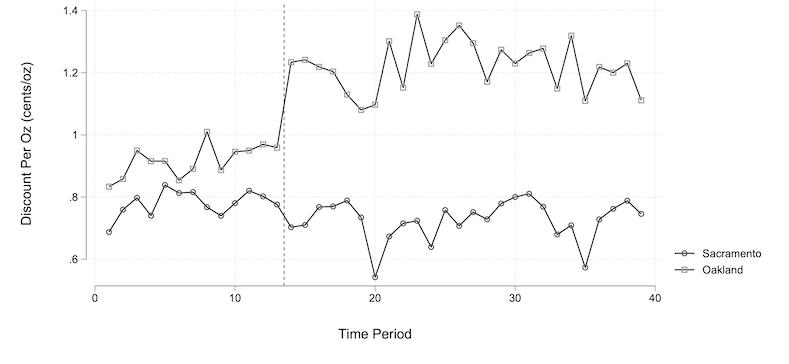

Supplement: S2 Fig — Each point represents a 4-week time period. July 1, 2017, the date of tax implementation, is represented by the dashed line. (TIF) [file pone.0285956.s002.tif]

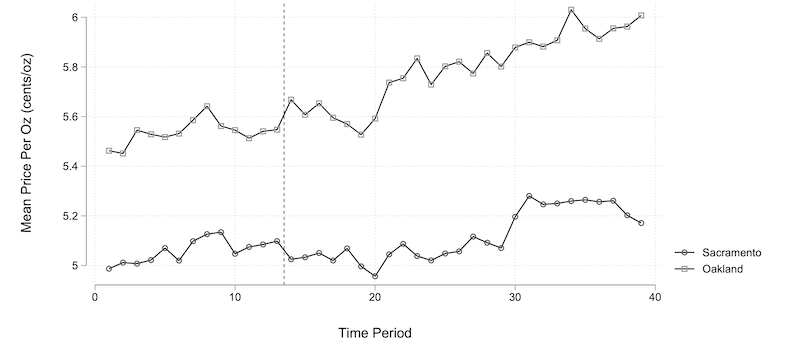

Supplement: S3 Fig — Each point represents a 4-week time period. July 1, 2017, the date of tax implementation, is represented by the dashed line. (TIF) [file pone.0285956.s003.tif]

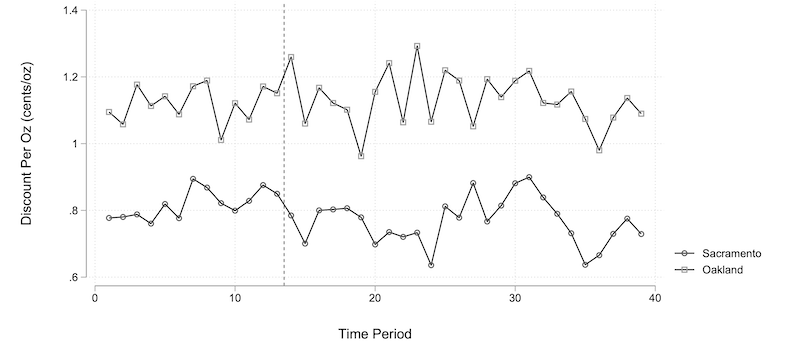

Supplement: S4 Fig — Each point represents a 4-week time period. July 1, 2017, the date of tax implementation, is represented by the dashed line. (TIF) [file pone.0285956.s004.tif]

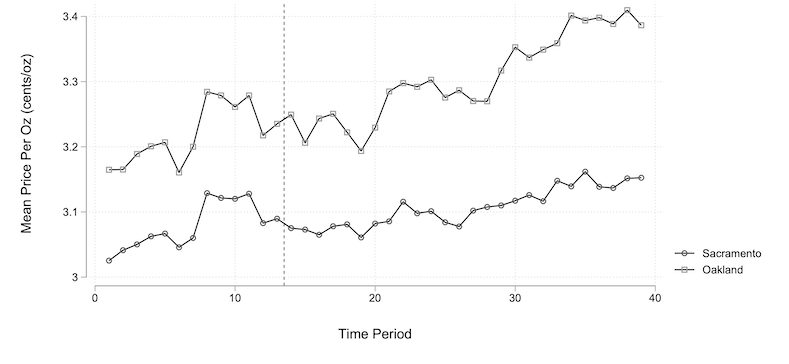

Supplement: S5 Fig — Each point represents a 4-week time period. July 1, 2017, the date of tax implementation, is represented by the dashed line. (TIF) [file pone.0285956.s005.tif]

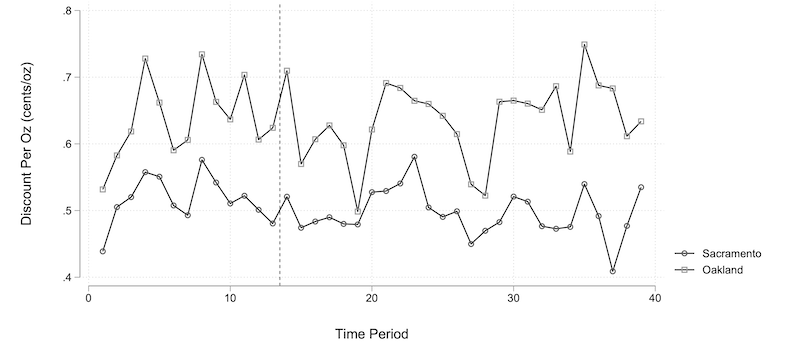

Supplement: S6 Fig — Each point represents a 4-week time period. July 1, 2017, the date of tax implementation, is represented by the dashed line. (TIF) [file pone.0285956.s006.tif]

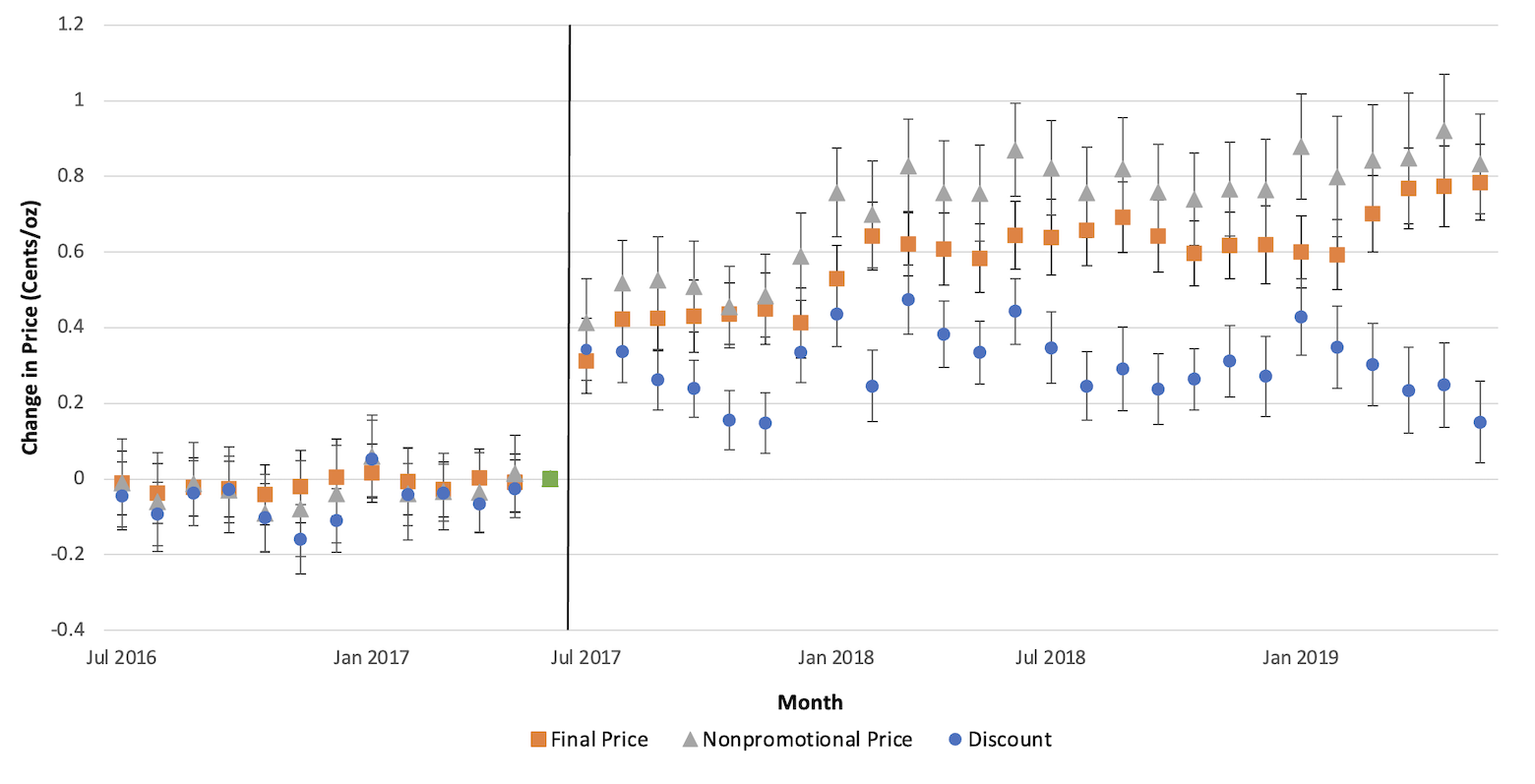

Supplement: S7 Fig — Each point represents an estimate from one time period from the event study approach. Time period 13 (June 4, 2017 –July 1, 2017) was used as the reference month and is represented in green. Bars represent the 95% confidence intervals. The solid line represents the tax implementation date. (TIF) [file pone.0285956.s007.tif]
