# Supplementary material for: The impact of the Oakland sugar-sweetened beverage tax on price promotions of sugar-sweetened and alternative beverages
Source: PLoS One. 2023 Jun 9;18(6):e0285956. doi: 10.1371/journal.pone.0285956 (PMC10256178; doi:10.1371/journal.pone.0285956)
Supplement: S1 Table — (DOCX) [file pone.0285956.s008.docx]

**S1 Table. Analytical Dataset Description**

| **Variable** | **Description** |
| --- | --- |
| site | Site (Oakland or Sacramento) |
| fu | Time point (baseline, 6-month, 12-month, or 24-month follow-up) |
| busid | Store identifier |
| productname | Beverage name |
| bev_sweet | Beverage type |
| oak_taxed | Indicator for whether the beverage was subject to the Oakland tax |
| IncomeTertile | Store income tertile (low-income, medium-income, or high-income) |
| actual_peroz | Final price in cents per ounce |
| peroz_reg | Nonpromotional price in cents per ounce |
| sale | Indicator for whether a sale was present |
| saledepth | Discount amount in cents per ounce |
