# Supplementary material for: The impact of the Oakland sugar-sweetened beverage tax on price promotions of sugar-sweetened and alternative beverages
Source: PLoS One. 2023 Jun 9;18(6):e0285956. doi: 10.1371/journal.pone.0285956 (PMC10256178; doi:10.1371/journal.pone.0285956)
Supplement: S1 File — (DOCX) [file pone.0285956.s010.docx]

To evaluate how the final price, nonpromotional price, and discount amount changed over time, an event study approach was used. This model is helpful to evaluate the parallel trends assumption and to see changes over time post-implementation. The model is:

$$\begin{aligned} {Outcome}_{ict}= \beta_{0}+ \sum_{t=1}^{12} \beta_{t}\delta_{t}Oakland_{c}+ \sum_{t=14}^{26} \beta_{t}\delta_{t}Oakland_{c}+ \beta_{27}Oakland_{c}+ \lambda_{i}+\delta_{t}+ \epsilon_{ict} \end{aligned}$$

where i represents the UPC, c represents the city, and t represents the time-period. Oakland_c_ is an indicator for whether the observation takes place in the city of Oakland. The parameters, β_1_ through β_12,_ represent the interaction of Oakland_c_ with the time period indicators leading up to the tax and the parameters, β_14_ through β_26,_ represent the interaction of Oakland_c_ with the time indicators after the tax. The last time period before implementation, δ_13_, was left out of the regression equation as a reference time period. This approach includes individual product fixed effects, represented by λ_i_, and time-period fixed effects represented by δ_t_.

S7 Fig shows the event study results for final price, nonpromotional price, and discount amount for SSBs over time with S1 Table providing the estimate values. As can be seen from the results, the difference between Sacramento and Oakland in each time period pre-tax was roughly zero for final price, nonpromotional price, and discount when controlling for UPC-level fixed effects and time-period fixed effects. This is supportive of the trends being parallel between Oakland and Sacramento in the pre-tax period. A level change can be seen visually at the point of implementation where final price and nonpromotional price increase and discount amount decreases. As is expected, the nonpromotional price remained consistently higher than the final price over time. There appears to be some seasonality to the discount depth after implementation with discounts being slightly deeper around January and slightly less deep around October and November. There does not appear to be a slope change in discount amount after the level change. Interestingly, as the nonpromotional price increased over two years, the discount amount did not increase proportionally.

**S1 Table. Event Study Estimates for Change in Final Price, Nonpromotional Price, And Discount Amount for Sugar-Sweetened Beverages**

| **Time Period** | **Final Price (cents per ounce)** | **Nonpromotional Price (cents per ounce)** | **Discount Amount (cents per ounce)** |
| --- | --- | --- | --- |
| 7/3/2016 - 7/30/2016 | -0.01 (-0.09, 0.07) | -0.01 (-0.1, 0.08) | -0.04 (-0.15, 0.07) |
| 7/31/2016 - 8/27/2016 | -0.04 (-0.12, 0.04) | -0.06 (-0.15, 0.02) | -0.09 (-0.2, 0.01) |
| 8/28/2016 - 9/24/2016 | -0.02 (-0.1, 0.06) | -0.01 (-0.1, 0.07) | -0.04 (-0.14, 0.07) |
| 9/25/2016 - 10/22/2016 | -0.03 (-0.1, 0.05) | -0.03 (-0.11, 0.05) | -0.03 (-0.12, 0.07) |
| 10/23/2016 - 11/19/2016 | -0.04 (-0.12, 0.04) | -0.09* (-0.17, -0.01) | -0.1 (-0.21, 0) |
| 11/20/2016 - 12/17/2016 | -0.02 (-0.11, 0.07) | -0.08 (-0.17, 0.02) | -0.16** (-0.27, -0.05) |
| 12/18/2016 - 1/14/2017 | 0 (-0.1, 0.11) | -0.04 (-0.14, 0.06) | -0.11* (-0.22, 0) |
| 1/15/2017 - 2/11/2017 | 0.02 (-0.06, 0.09) | 0.06 (-0.03, 0.15) | 0.05 (-0.06, 0.16) |
| 2/12/2017 - 3/11/2017 | -0.01 (-0.09, 0.08) | -0.04 (-0.12, 0.05) | -0.04 (-0.14, 0.06) |
| 3/12/2017 - 4/8/2017 | -0.03 (-0.1, 0.04) | -0.03 (-0.12, 0.06) | -0.04 (-0.14, 0.07) |
| 4/9/2017 - 5/6/2017 | 0 (-0.07, 0.08) | -0.04 (-0.11, 0.04) | -0.07 (-0.17, 0.04) |
| 5/7/2017 - 6/3/2017 | -0.01 (-0.09, 0.07) | 0.01 (-0.08, 0.1) | -0.03 (-0.13, 0.07) |
| 6/4/2017 - 7/1/2017 | REF | REF | REF |
| 7/2/2017 - 7/29/2017 | 0.31*** (0.23, 0.4) | 0.41*** (0.33, 0.5) | 0.34*** (0.23, 0.46) |
| 7/30/2017 - 8/26/2017 | 0.42*** (0.33, 0.51) | 0.52*** (0.43, 0.61) | 0.34*** (0.21, 0.47) |
| 8/27/2017 - 9/23/2017 | 0.42*** (0.34, 0.51) | 0.53*** (0.43, 0.62) | 0.26*** (0.14, 0.39) |
| 9/24/2017 - 10/21/2017 | 0.43*** (0.33, 0.53) | 0.51*** (0.42, 0.6) | 0.24*** (0.11, 0.37) |
| 10/22/2017 - 11/18/2017 | 0.44*** (0.36, 0.52) | 0.46*** (0.37, 0.54) | 0.16* (0.03, 0.28) |
| 11/19/2017 - 12/16/2017 | 0.45*** (0.36, 0.54) | 0.48*** (0.39, 0.58) | 0.15* (0.02, 0.28) |
| 12/17/2017 - 1/13/2018 | 0.41*** (0.32, 0.51) | 0.59*** (0.49, 0.69) | 0.34*** (0.2, 0.47) |
| 1/14/2018 - 2/10/2018 | 0.53*** (0.44, 0.62) | 0.76*** (0.66, 0.85) | 0.44*** (0.31, 0.56) |
| 2/11/2018 - 3/10/2018 | 0.64*** (0.55, 0.73) | 0.7*** (0.62, 0.78) | 0.25*** (0.13, 0.36) |
| 3/11/2018 - 4/7/2018 | 0.62*** (0.54, 0.71) | 0.83*** (0.74, 0.91) | 0.47*** (0.36, 0.59) |
| 4/8/2018 - 5/5/2018 | 0.61*** (0.51, 0.7) | 0.76*** (0.67, 0.85) | 0.38*** (0.24, 0.53) |
| 5/6/2018 - 6/2/2018 | 0.58*** (0.49, 0.68) | 0.76*** (0.66, 0.85) | 0.33*** (0.21, 0.46) |
| 6/3/2018 - 6/30/2018 | 0.64*** (0.55, 0.73) | 0.87*** (0.78, 0.96) | 0.44*** (0.33, 0.56) |
| 7/1/2018 - 7/28/2018 | 0.64*** (0.54, 0.74) | 0.82*** (0.74, 0.91) | 0.35*** (0.22, 0.48) |
| 7/29/2018 - 8/25/2018 | 0.66*** (0.56, 0.75) | 0.76*** (0.66, 0.85) | 0.25*** (0.11, 0.38) |
| 8/26/2018 - 9/22/2018 | 0.69*** (0.6, 0.79) | 0.82*** (0.72, 0.92) | 0.29*** (0.15, 0.44) |
| 9/23/2018 - 10/20/2018 | 0.64*** (0.55, 0.74) | 0.76*** (0.66, 0.85) | 0.24** (0.1, 0.38) |
| 10/21/2018 - 11/17/2018 | 0.6*** (0.51, 0.68) | 0.74*** (0.65, 0.83) | 0.26*** (0.13, 0.4) |
| 11/18/2018 - 12/15/2018 | 0.62*** (0.53, 0.71) | 0.77*** (0.68, 0.86) | 0.31*** (0.19, 0.43) |
| 12/16/2018 - 1/12/2019 | 0.62*** (0.52, 0.72) | 0.76*** (0.66, 0.86) | 0.27*** (0.15, 0.4) |
| 1/13/2019 - 2/9/2019 | 0.6*** (0.5, 0.7) | 0.88*** (0.77, 0.99) | 0.43*** (0.3, 0.55) |
| 2/10/2019 - 3/9/2019 | 0.59*** (0.5, 0.69) | 0.8*** (0.68, 0.92) | 0.35*** (0.2, 0.49) |
| 3/10/2019 - 4/6/2019 | 0.7*** (0.6, 0.8) | 0.84*** (0.74, 0.95) | 0.3*** (0.17, 0.44) |
| 4/7/2019 - 5/4/2019 | 0.77*** (0.66, 0.87) | 0.85*** (0.74, 0.95) | 0.23*** (0.11, 0.36) |
| 5/5/2019 - 6/1/2019 | 0.77*** (0.67, 0.88) | 0.92*** (0.81, 1.03) | 0.25*** (0.11, 0.39) |
| 6/2/2019 - 6/29/2019 | 0.78*** (0.68, 0.88) | 0.83*** (0.73, 0.94) | 0.15* (0.02, 0.28) |

Linear regression event study models compared Oakland and Sacramento in each time period pre- and post-implementation for each of the beverage types. Time period 13 (June 4, 2017 – July 1, 2017) was used as the reference month before implementation (heavy gridline). Each estimate represents estimated change in price in that time period relative to the reference time period. Parentheses include 95% confidence intervals. REF = reference time period. * p<0.05; ** p<0.01; *** p<0.001
